# Healthy Places for Children: The Critical Role of Engagement, Common Vision, and Collaboration

**DOI:** 10.3390/ijerph17249277

**Published:** 2020-12-11

**Authors:** Mildred E. Warner, Xue Zhang

**Affiliations:** 1 Department of City and Regional Planning, Cornell University, Ithaca, NY 14850, USA; xz435@cornell.edu; 2 Department of Global Development, Cornell University, Ithaca, NY 14850, USA

**Keywords:** child-friendly communities, zoning codes, services, collaboration, engagement, common vision

## Abstract

Planning plays a critical role in promoting healthy communities for children. We conducted a national survey of United States (US) cities and counties in 2019 and found only half of the 1312 responding communities report they give attention to the needs of children in their community plans. Those that do, provide more services and have more child-friendly zoning codes. We use a human ecological framework to build structural equation models of child-friendly zoning and services. We find communities with more engagement of families with children and youth and a common vision across generational, race, and ethnic lines report higher levels of child-friendly zoning and services. Collaboration between health providers and schools builds trust and leads to more services. However, child-friendly zoning is lower in communities with higher child poverty, and in suburbs and rural areas. Our results support a dynamic human ecological model where the processes of collaboration, inclusion, and engagement are key to creating healthy places for children. These processes may be especially important in addressing the unique challenges of suburban and rural communities.

## 1. Introduction

In August 2020, UNICEF USA launched its Child Friendly Cities Initiative in the United States (US) with Houston, TX; San Francisco, CA; Minneapolis, MN, and Prince George’s County, MD. UNICEF defines a child-friendly city as “a city or community where the voices, needs, priorities and rights of children are an integral part of public policies, programmes and decisions” [1] (p. 10). While these cities constitute the first cohort of cities collaborating with UNICEF, many cities have branded themselves child or family friendly in the past. A 2008 survey of 900 planners across the US found that 40% reported their community was “family-friendly” [2]. A 2013 survey of cities and counties found only 39% considered the needs of children in their community plans [3]. We updated the survey in 2019 and found little improvement; 40% of communities reported addressing the needs of children in their comprehensive plans [4]. UNICEF reports that the integration of local government planning is key to sustain Child Friendly Cities initiatives [1].

In this paper, we examine the role of local government in promoting public health by creating more child-friendly zoning codes and services. We give attention to the impact of an inclusive social environment, which incorporates engagement of families with children, attitudes towards families with children, and a common vision on planning for all ages in a diverse community. Using a human ecological theory framework [5], we designed a national survey in 2019 called Planning for All Ages to examine factors related to child-friendly zoning codes and services across US municipalities. 1312 US municipalities responded. This survey enables us to explore three research questions: (1) What role do health providers and schools play in building child-friendly communities? (2) What is the impact of social inclusion, planning, and engagement on child-friendly zoning and services? (3) Do child-friendly zoning codes and services vary across communities with different demographic characteristics? Our results support a dynamic human ecological model where social inclusion and engagement link the micro (family), meso (service provider), and macro (planning and zoning) systems. By integrating the physical, service, and social engagement layers, communities are able to build more healthy places for children. This dynamic approach offers promise for communities across the rural–urban spectrum.

## 2. Literature Review

What makes a healthy community for children? Public health looks beyond the individual and pays attention to the social determinants of health, and the physical and social environment of the community where people live, work, and play [6,7,8,9]. UNICEF describes a child-friendly city as a place which offers a safe environment, access to essential services, and the inclusion and participation of children [1]. To address the social determinants of children’s health requires specific attention to the built environment and services. This is where local governments play a key role in planning, service delivery, and collaboration with health-related agencies.

Public health often uses a human ecology approach [5,10,11] to show the nested nature of individual health in family, community, and policy systems. This approach has been used to structure community level studies of public health as it relates to early childhood [6,12,13] and family wellbeing [14,15,16], and to design comprehensive models for community action and policy engagement [6,7,10,11]. A human ecological model of public health looks at multiple layers: the community built environment, community level services, public engagement, collaboration among health related agencies, and geographic differences across place [17].

The built environment matters. WHO’s [18] report on children’s health shows the important linkage between the community level built environment (streets, neighborhoods, and housing) and child health outcomes, such as respiratory infections, diarrheal diseases, injuries, and cancer. To create better built environments for children requires zoning codes that give attention to children’s needs [19]. Community planning provides the policy foundation for zoning codes and services which meet children’s needs [20,21]. Most studies focus on the relation between the physical built environment and child health, such as streets and sidewalks to ensure walkability [8,22,23], neighborhood characteristics such as mixed use and access to play spaces [9,24,25,26,27,28], and housing characteristics [13,29].

Services matter. A range of community services are key to children’s health—adequate quality child care [30], community centers, and play spaces [9,31], family support programs [15], and access to nutrition and health services [32]. Increasingly, health studies recognize the need to identify which agencies are trusted by families with children, and which ones they turn to for information [33]. Collaboration with schools is increasingly recognized as a means to reach children and address their public health needs [34,35,36]. Schools can provide access to health care, recreation and nutrition for children. Cross agency collaboration has been identified as a missing domain in WHO’s age-friendly planning directives [33,37]. Collaboration is especially important between schools and other health related local government agencies [34]. Health providers can reach out to neighborhoods and build collaborations to address racial and health disparities among children [38].

Engagement matters. Community attitudes, vision, and opportunities for engagement are key to building more age-friendly communities to support public health [39]. Healthy cities for children create opportunities for participation and community inclusion [8,35,40]. Engagement and inclusion can improve the range of services provided in the community [21,41], and promote better health outcomes for children [42,43,44].

Cross-agency collaboration matters. Public health agencies such as the Robert Wood Johnson Foundation and the National Institutes for Health Disparities recognize the importance of engagement and inclusion in meeting the needs of children [10,11]. Cross-agency collaboration, local government policy and social inclusion are recognized as key community level factors determining public health outcomes [39,41]. Planners have an especially important role in promoting child health in both rural and urban settings [45,46,47].

Geography matters. Health disparities are greatest in low income and minority communities in the US [7]. Disparities are also higher in rural communities [41,48]. Children lack voice and require more responsive community boards to recognize their needs. But US local government boards are often dominated by older residents, who may be less responsive to the needs of children [3]. Political barriers and motivators are important challenges communities must overcome to address health disparities among children. While engagement is key, so too is a culture of inclusion [49].

**Figure 1:**
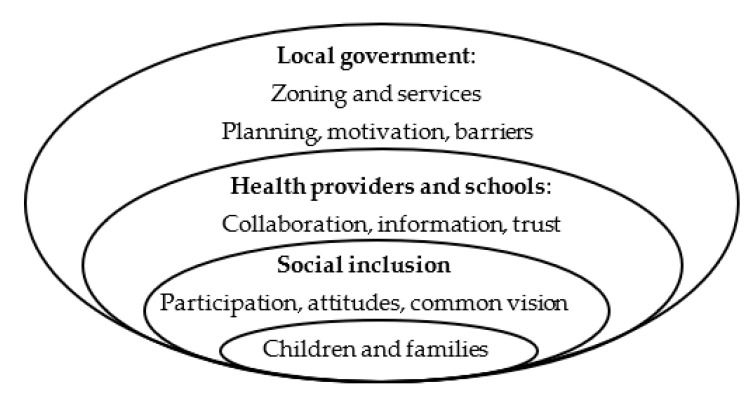
Research framework-healthy places for children.

This study addresses a critical gap in the literature—the role of local government in improving child health through planning, zoning and services. We give special attention to participation and inclusion, and we measure collaboration across health-related agencies at the community level. Our human ecological approach provides a framework to assess the social determinants of health and the role of community level institutions in creating healthy places for children. We explore how the different layers in the human ecology framework (Figure 1) are related to build healthy places for children.

## 3. Method

### 3.1. Research Questions

We built a unique data set to address our research questions. We designed and conducted a national Planning for All Ages survey in 2019 to explore local government actions to build child-friendly communities. In addition to data on planning and zoning for the built environment, our survey includes unique measures of social inclusion: children’s participation, community attitudes, and a common vision. Our survey also provides a rich dataset on health-related agencies in child-friendly communities. This includes their role in partnering with local government to deliver services, providing information on services, and building trust with children and families. Our data enable us to articulate a human ecological framework which explores factors related to the social determinants of children’s health, specifically, child friendly zoning codes and services. Three research questions are addressed: (1) What role do health providers and schools play in building child-friendly communities? (2) What is the impact of social inclusion, planning and engagement on child-friendly zoning and services? (3) Do child-friendly zoning codes and services vary across communities with different demographic characteristics?

### 3.2. Data

Our data is drawn from the Planning for All Ages survey we conducted in 2019. We collaborated with the International City/County Management Association (ICMA) to send the survey to city and county managers across the US. The sample frame included all counties and all municipalities over a population of 25,000, and a one in three sample of municipalities under 25,000, and a one-in-2.5 sample of towns and townships over 2500 in population for a total of 8016 local governments. 1312 places responded for a response rate of 16%. Two-sample Kolmogorov–Smirnov test shows the sample is representative by geography but captures more larger places than rural communities.

The survey included more than 100 questions (yes/no, Likert scale) on factors related to building a livable community for children. Survey questions covered local governments’ actions on child-friendly planning, zoning codes (measuring level of coverage), services, collaboration with health-related agencies, and social inclusion. Questions on barriers, motivators, and political pressure were also included. The majority of respondents were chief administrative officers (55%) or planners (20%). We linked the survey data with socioeconomic data from the American Community Survey (2009–2013, 2014–2018) to capture child socioeconomic conditions and community demographic structure.

### 3.3. Measures

#### 3.3.1. Outcome Variables: Community Child-Friendly Zoning Codes and Services

This study explores factors related to healthy places for children. The Planning for All Ages survey provides a rich dataset on the level of coverage of child-friendly zoning codes and the number of child-focused services at the community level. These are the two dependent variables in our analysis: child-friendly zoning codes and services.

● Child-friendly Zoning Codes

The zoning codes variable measures the proportion of the community covered by fourteen child-friendly features related to neighborhood, street and housing characteristics. The respondents assessed the percent of their community covered by each zoning code on a scale of 1 to 5 (0% = 1, 0–25% = 2, 25–50% = 3, 50–75% = 4, ≥75% = 5). The zoning code variable aggregates the scale (1 to 5) of community coverage for each of 14 zoning codes to measure the level of the community covered by those codes. The zoning code elements are shown in Table 1.

The most common zoning code is “allow family-sized housing (with 2 or more bedrooms)”. More than 50% of respondents reported that at least 75% of their community was covered by zoning codes requiring family-sized housing. Access to outdoor recreation and open space is also a child-friendly feature provided by most communities. More than half of respondents indicated that at least 50% of their communities are covered by zoning codes which “promote parks or recreation facilities in all neighborhoods”. However, most child-friendly zoning codes only covered between a quarter and a half of the community; especially the codes designed to improve children’s independent mobility and those promoting mixed use neighborhoods. These codes include: “mandate sidewalk system”, “contain pedestrian-friendly design guidelines”, “require street connections between adjacent developments”, “allow multi-family housing”, “allow child care centers”, and “allow child care business in residential units by right”. The zoning codes with less than 25% coverage are those that “require complete streets”, “promote affordable housing”, “allow accessory dwelling units”, and “allow mixed-use (e.g., retail and services in residential areas)”. More than half of communities lack two zoning codes: “mandate universal design for new housing construction (physically accessible to people with limited mobility)”, and “provide density bonuses (e.g., for affordable housing, open space, transit)”.

● Child-focused Services

Our survey measured the number of child-focused services available in the community and through public schools. See Table 2. Nine community-provided services and four school-provided services were measured. The most common community-provided services are after-school programs (72%), and summer camps (67%). Other education and training services were less common, including publicly supported preschool (47%), youth employment programs (40%), and family literacy/parenting programs (40%). About a third of communities have a youth center (37%) and walk-to-school programs (31%). The least common services in communities are business assistance, loans and grants to support childcare (16%), and home visiting for families with children (16%).

The survey asked about four health and recreation services provided in public schools. Less than half of the respondents reported their schools provided these services. The most common school-provided service was “child nutrition for evenings/weekends or summer,” reported by 42% of respondents. Thirty two percent of communities reported their schools provide “childcare services.” This is twice the level of community-provided childcare (16%). Thirty one percent of communities report their schools provide “recreation programs for all ages.” However, school based “health care services for all ages” was only reported by 11% of communities.

#### 3.3.2. Independent Variables

Our independent variables address three layers in our human ecological model: macro system—local government policy and planning, meso system—health related agencies and collaboration, and micro system—child characteristics and participation. Descriptive statistics are shown in Table 3.

● Local government: planning, motivators, political orientation, and barriers

*Planning*. The survey asked if the community addresses the needs of children in the comprehensive plan, the economic development plan, and the transportation plan. Less than half of communities do. Our planning index is composed of five elements. Forty percent of communities report their comprehensive plan addresses the “needs of families with children,” and 31% report their comprehensive plan addresses “schools or school siting.” Only 29% of communities consider the “mobility needs of children” in their transportation plan. The “needs of children” are even less likely to be included in community economic development plans (16% of respondents), and only 11% of economic development plans addressed the “childcare needs of parents.” We expect that communities which pay more attention to the needs of children in their plans will have more child-friendly zoning codes and services.

*Motivation/Pressure*. The survey asked what motivates communities to engage in planning for children. More than half of the respondents reported that they were motivated by the “desire to attract or retain children in the community” (52%), the “availability of government funding for services or programs” (52%), and “interest of staff or prioritization by staff” (51%). Designation as an “Age Friendly” or “Livable Community for all Ages” was listed as a motivation by 40% of communities. A higher percent of communities reported pressure from “local elected leaders” (38%), and the “political engagement of families with children” (36%), than pressure from “business/nonprofit leaders” (28%). We built an index of these seven elements for our variable on motivation/pressure.

*Political orientation.* We explore if the political orientation of the local governing board is related to child-friendly zoning and services. Forty-four percent of communities report their local governing board is predominantly conservative, compared to 11% reporting their governing board is liberal. Forty-five percent report their governing board is evenly mixed. We create a dummy variable to measure if the community has a conservative governing board.

*Barriers*. Our survey measured twelve barriers to joint programing for different ages, which we aggregated into an index. The most common barriers reported were funding and information: “lack of funding” (65%), “segregated funding streams” (41%), “lack of information” (38%), “liability” (31%), and “lack of common data systems” (25%). Regulation and opposition were less commonly reported as barriers: “regulations to protect children” (13%), “regulations to protect frail elders” (8%), “elected official opposition” (7%), and “department head or staff opposition” (5%). Other barriers included “turf issues” (22%), “customer preference for age-segregated services” (18%), and “concerns about safety” (16%).

● Health providers and schools: collaboration in serving families with children

Collaboration plays an important role in service delivery for all ages [33]. We measure community cross-agency collaboration with four key service providers for children: (1) the public health department, (2) hospital or health care providers, (3) schools, and (4) childcare resource and referral agencies. We differentiate collaboration into three elements: cross-agency partnerships to serve families with children, information delivery to families with children, and the level of trust these institutions enjoy among families with children.

*Partnership* measures the number of health-related agencies which engage in cross-agency partnership with local government to serve families with children. Among the four institutions, school districts were the most likely to engage in partnerships with local government (57% of responding communities). Partnership with the public health department was reported by 44% of communities, partnership with hospital or health care providers was reported by 38% of communities, and partnership with childcare resource and referral agencies was reported by 37% of communities.

*Information delivery* measures if local governments work with schools and health care providers to deliver information and services to families with children. Local governments commonly work with schools (66%), compared to only 32% of the respondents who reported their local governments work with health care providers.

*Trust* measures if families with children trust schools or health care providers for information about services. Eighty-one percent of the respondents reported that families with children trust schools for information, and 49% of the respondents indicated that families with children trust health care providers.

● Social Inclusion

Inclusion is key to designing livable communities [1,42,49,50]. In this study, social inclusion is measured by public engagement, attitudes and a common vision. Each variable is an aggregate index of survey responses.

*Engagement.* Our survey asked about the level of engagement of families with children and youth in planning for their needs. The engagement of each group was measured on a three-point scale (1 = not at all engaged, 2 = somewhat engaged, and 3 = very engaged). The level of engagement is higher for families with children than for youth. Two-thirds of families with children are somewhat engaged and 12% are very engaged. This is higher than youth, where only 48% of communities report youth are somewhat engaged, and only 6% report that youth are highly engaged. We expect communities with a higher level of engagement will have more child-friendly zoning and services.

*Attitudes*. Our survey asked about community attitudes toward families with children. Each question is on a scale from strongly disagree (1), to neutral (3), to strongly agree (5). Attitudes includes five elements. More than half of the respondents agreed that “children are a resource for the community” (91% agree), “the community has a responsibility to care for children and youth” (84% agree), “the needs of families with young children are similar to the needs of the elderly with regards to the physical environment (e.g., walkability, parks, transportation, affordable housing)” (76% agree), and “families with children represent a valuable consumer population” (90% agree). Most respondents were neutral about whether “families with children generate sufficient tax revenue to cover the cost of services they demand” (22% agree).

*Common vision* includes three elements: “participation of families with children has led to a common vision regarding planning for all ages” (37% agree), “my community is not divided by race, class, or old-timer/newcomer divisions” (35% agree), and “ethnic or cultural diversity has led to new approaches to planning or programming for all ages” (37% agree). Most respondents were neutral about a common vision in their communities, compared to a much higher percentage of communities reporting positive attitudes toward children.

● Children and families: socio-economic conditions and demographic structure

We control for child socioeconomic conditions: percent of population under age 18, population growth of children under 18, and the child poverty rate. Other demographic variables include population, population density, and percent White.

We classify communities as metro core, suburb, and rural based on US Census delineations. Metro core places have at least one principal city and suburbs are other places inside metropolitan areas. Rural are nonmetropolitan places. Metro core places are set as the reference group.

### 3.4. Analysis

We use our unique national survey to examine factors related to healthy places for children. We use linear regression to assess the relation between child-friendly features (zoning codes and services), and the other layers in our human ecological model: macro system—local government (planning and political orientation), meso-system—cross-agency collaboration, and micro system—child characteristics. We give special attention to social inclusion and participation as mechanisms to encourage healthy places for children. The services model also includes collaboration and trust among health providers and schools. The model is run in STATA 14.0, and uses maximum likelihood in Structural Equation Modeling to address missing values [51]. We expect more services for children will be associated with collaboration between health providers and schools (Research question 1); a positive relation will be found between child-friendly features and planning and social inclusion (Research question 2), and child-friendly features will vary by geography and demographic characteristics (Research question 3).

## 4. Results

The model results are shown in Table 4. To compare the marginal effects of variables, the coefficients are standardized. The results of the services model show the important role of collaborating with health providers and schools in delivering services for children (Research question 1). Cross-agency partnership has the largest effect on the number child-focused services in the community (β = 0.23). Information and trust also matter (β_information_ = 0.20, β_trust_ = 0.09). Collaboration between local government and health-related agencies in delivering services, disseminating information, and building trust are associated with communities that offer more child-focused services.

Variables associated with planning and social inclusion address research question 2. Child-friendly features, both zoning codes and services, are more common in communities which have plans to address the needs of children and which encourage a higher level of engagement of families with children and youth. These communities also face more motivation/pressure to engage in planning and they are more likely to report a common vision. Planning has the largest effect on the number of child-friendly services. The results show that communities which offer more services for children consider the needs of children in multiple plans (β = 0.12), and community planners feel more motivation/pressure to engage in planning for children (β = 0.09). Communities with conservative boards are not related to child-friendly features. The number of barriers is higher in communities with more services (β = 0.05) but is not related to zoning codes.

Social inclusion matters for child-friendly features. The engagement of families with children and youth has a similar positive effect on child-friendly zoning codes as including attention to children in formal community plans (β_engagement_ = 0.13, β_planning_ = 0.12). A common vision across generational, race and ethnic lines has a similar positive effect as engagement on both zoning codes and services. Attitudes are not related to child-friendly features.

Coefficients of child socioeconomic conditions and demographic structure address research question 3. Community services for children are not limited by the socioeconomic conditions of children in the community. Zoning codes, by contrast, are higher in communities with more children (β = 0.08), but places with a higher child poverty rate have a lower level of child-friendly zoning codes (β = −0.10). The results of demographic structure show that larger and denser communities have more child-friendly features. Population density has the largest effect on zoning codes (β = 0.38). Compared to the metro core, suburbs have fewer child-friendly zoning codes (β = −0.13) and services (β = −0.12). Rural areas also lag in services for children (β = −0.10).

## 5. Discussion

This study uses a unique national survey to explore factors related to healthy places for children. Our first research question examines the role of health-related institutions on services for children, and finds collaboration between local governments, healthcare providers, and schools is higher in communities that offer more child-focused services. This shows the importance of cross-agency collaboration, especially joint service delivery with schools. Previous studies have found that joint use with schools can enhance community health by increasing access to recreation, literacy and nutrition services [7,31,38,52]. In communities that provide more services for children, local governments partner with schools to deliver information and services; and schools are trusted by families with children. Cross-agency collaboration has been identified as the missing domain in UNICEF’s child friendly framework and WHO’s age friendly framework [33]. This study confirms the importance of collaboration with schools to build healthy places for children.

Our second research question explores the role of planning and social inclusion on child-friendly zoning codes and services. While prior studies have identified the critical role of engagement of families with children and youth in promoting community health [3,19,39,42,49], our results show that a common vision across generations, race, and ethnicity is as important as engagement in building healthy communities for children. Positive attitudes toward families with children are not enough. They need to be articulated in community plans through processes of engagement [19]. Social inclusion is more than engagement, it includes trust and a common vision of planning for all ages in a diverse community. Future research should explore how engagement leads to more common vision.

**Figure 2:**
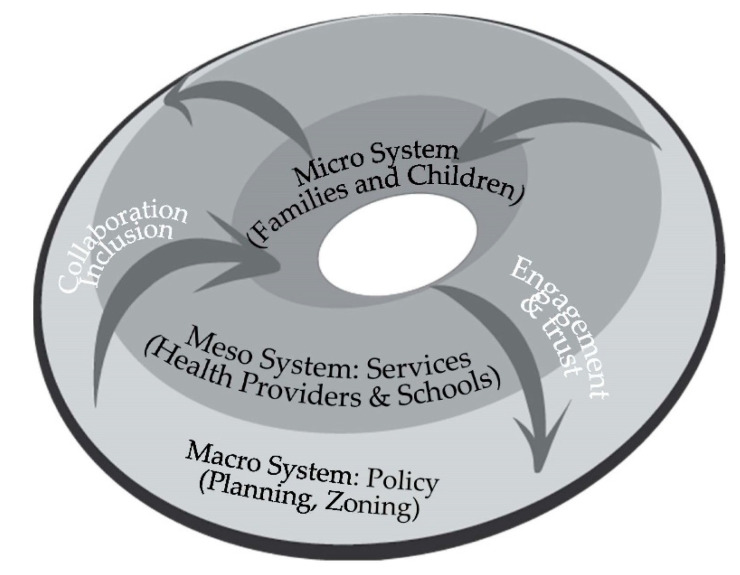
A Dynamic Human Ecology Framework: Healthy Places for Children.

Our study makes an important contribution to human ecological theory. While prior studies have articulated human ecology as a hierarchy of institutional layers that affect child welfare [5,12], we see a more dynamic process. See Figure 2. Our models explore the dynamics between the micro layer of children and families, the meso layer of services, and the macro layer of policy (zoning, planning). In this dynamic model, local governments, through collaboration and inclusion, coordinate with the meso system of service providers to reach the micro system of children and families. But this is not a one-way process. Through engagement and trust, children and families can reach up from the micro layer to the meso system of service providers, and affect macro policies of planning, zoning, and service delivery. Such dynamic models of active citizenship have been shown to enhance family wellbeing in other international contexts [14,16].

Child-friendly zoning codes and services are differentiated by child socioeconomic conditions and geography (Research question 3). Child-friendly zoning codes are more likely in communities with more children, but less likely in communities with more child poverty. The level of services, by contrast, is not differentiated by need. This suggests that zoning needs to be more responsive to the needs of children and families. While metro core places have more child-friendly zoning codes and services, suburbs and rural areas lag. Denser communities are more likely to be child friendly, but this is mostly a metro core effect, as metro core areas have higher population density (2521 per sq. Mile) than suburbs (1926 per sq. Mile) and rural communities (470 per sq. Mile). Our findings show disparities in healthy places for children across the urban-rural divide: suburban areas lag in zoning codes, and suburbs and rural areas lag in services. These geographic disparities raise special challenges for creating healthy places for children in suburbs and rural communities. Both UNICEF’s and WHO’s initiatives are focused on cities. More attention needs to be given to how to promote more healthy environments for children in suburbs and rural communities, especially the links to planning and community development [3,46,53]. Suburban places have a higher percentage of children (23.20%) than metro core (21.75%) and rural (21.98%), but a lower level of child-friendly zoning codes and services. Rural areas have higher child poverty (22.07%) than metro core (21.06%) and suburbs (13.25%), so the negative relation between poverty and zoning is primarily a rural effect.

Planning needs to give more attention to policies which create healthy places for children in suburban and rural communities. Mixed use and walkable neighborhoods are harder to implement in rural areas. Where physical design and density limit age-friendly approaches, attention can be given to the social layer. Through engagement from below and inclusion from above, governments and residents can work together to identify new approaches to addressing the needs of children. Intergenerational approaches offer promise through shared services in community centers and schools, changing norms and incorporation of informal networks with formal planning and service provision [47,54].

This study has several limitations. First, the data on child-friendly features, collaboration, planning, and social inclusion are from a national survey sent to local government managers. While the survey distinguished many elements of practice (zoning and services), qualitative research is needed to get inside the dynamics of process in a community setting. We are able to show that engagement, a common vision and planning all have similar relationships to child-friendly community features. However, we do not know how these process elements work together. That would require more qualitative research. Future case study research should explore community development strategies encouraging engagement, promoting cross-agency collaboration, and creating a common vision on planning for all ages.

## 6. Conclusions

Child health is an increasing concern for planners, as it should be. Our 2019 national survey shows only 40% of US communities address the needs of children in their comprehensive plans. Zoning codes lay the foundation for future development, but child-friendly zoning codes are less common in suburbs and in communities with higher child poverty. This raises concerns about how to address the social determinants of health in communities where children need it the most. Social inclusion plays a major role, as does collaboration between schools, health agencies, and the local government. While planning has given attention to the physical environment, our study shows the importance of social inclusion—to connect the individual, family, service provider and local government levels. Social inclusion works in both directions—as governments reach down to providers and families, but also as families with children reach up to effect change in the systems that serve them at the meso and macro levels. Engagement, trust, inclusion, and collaboration—these are the social keys to healthy places for children.

## Figures and Tables

**Table 1:** Child-friendly Zoning Codes.

**Child-Friendly Zoning Code Index:** Does your community have zoning, subdivision regulations, or building codes? If yes, in what percent of your community do those regulations do the following? Code: 0% = 1, 0–25% = 2, 25–50% = 3, 50–75% = 4, ≥75% = 5. Median values are shown in parentheses below.
● Allow family-sized housing (with 2 or more bedrooms) (5)
● Promote parks or recreation facilities in all neighborhoods (4)
● Mandate sidewalk system (3)
● Contain pedestrian-friendly design guidelines (3)
● Require street connections between adjacent developments (3)
● Allow multi-family housing (3)
● Allow childcare centers (3)
● Allow childcare business in residential units by right (3)
● Require “complete streets” (2)
● Promote affordable housing (2)
● Allow accessory dwelling units (2)
● Allow mixed-use (e.g., retail and services in residential areas) (2)
● Mandate universal design for new housing construction (physically accessible to people with limited mobility) (1)
● Provide density bonuses (e.g., for affordable housing, open space, transit) (1)

Data source: Planning for all ages survey, 2019. *n* = 1312 US municipalities and counties.

**Table 2:** Child-focused Services.

**Child-Focused Services:** Which of the following facilities, services or programs are available in your community? (% yes)
● After-school programs (72%)
● Summer camps (67%)
● Publicly supported preschool (47%)
● Youth employment programs (40%)
● Family literacy/parenting programs (40%)
● Youth center (37%)
● Walk-to-school programs (31%)
● Business assistance, loans and grants to support child care (16%)
● Home visiting for families with children (16%)
Which of the following services are offered in your community’s public schools? (% yes)
● Child nutrition for evenings/weekends or summer (42%)
● Childcare services (32%)
● Recreation programs for all ages (31%)
● Health care services for all ages (11%)

Data source: Planning for all ages survey, 2019. *n* = 1312 US municipalities and counties.

**Table 3:** Planning Healthy Places for Children: descriptive statistics.

Variables	Obs.	Mean	Std. Dev.	Min.	Max.
Child-friendly features					
Child-friendly zoning codes (14 elements, scale 1–5)	1011	41.44	12.38	14	70
Child-focused services (13 elements)	1312	4.83	3.09	0	13
Planning and political orientation					
Planning (5 elements) ^1^	1264	1.29	1.37	0	5
Motivation/Pressure (7 elements) ^1^	1312	2.96	2.13	0	7
Conservative governing board (1 = yes) ^1^	1154	0.44	0.50	0	1
Barriers (12 elements) ^1^	1312	2.94	2.27	0	12
Collaboration with health-related agencies					
Partnership (4 elements) ^1^	1312	1.56	1.27	0	4
Information (2 elements) ^1^	1312	0.99	0.77	0	2
Trust (2 elements) ^1^	1312	1.30	0.73	0	2
Social Inclusion					
Engagement (2 elements, scale 1–3) ^1^	1243	3.52	1.01	2	6
Attitudes (5 elements, scale 1–5) ^1^	1202	19.53	2.77	6	25
Common vision (3 elements, scale 1–5) ^1^	1208	9.35	2.03	3	15
Child socioeconomic conditions					
Percent population under 18 (%) ^2^	1312	22.57	4.74	3.05	45.99
Population growth under 18 (%) ^2,3^	1311	−0.87	12.97	−51.04	96.06
Poverty rate under 18 (%) ^2^	1312	17.34	11.95	0	76.73
Demographic structure					
Percent White population (%) ^2^	1312	81.71	17.13	5.95	100.00
Population (ln) ^2^	1312	9.94	1.45	5.98	15.19
Population density (ln) ^2^	1312	6.17	2.06	−0.82	10.87
Metro core (1 = yes, reference group) ^4^	1312	0.18	0.38	0	1
Suburb (1 = yes) ^4^	1312	0.52	0.50	0	1
Rural (1 = yes) ^4^	1312	0.31	0.46	0	1

Data sources: ^1^ Planning for all ages survey 2019, ^2^ American Community Survey 2014–2018, ^3^ American Community Survey 2009–2013, ^4^ Census Bureau Metro-Micro Delineation files Sept. 2018.

**Table 4:** Factors related to child-friendly zoning codes and services: SEM model results.

	Child-Friendly Features			
	Zoning Codes ^1^		Services ^1^	
	Std. Coef.	S.E.	Std. Coef.	S.E.
Collaboration with health-related agencies				
Partnership (4 elements) ^1^	-	-	0.232 **	(0.06)
Information (2 elements) ^1^	-	-	0.202 **	(0.10)
Trust (2 elements) ^1^	-	-	0.093 **	(0.10)
Planning and political orientation				
Planning (5 elements) ^1^	0.119 **	(0.26)	0.123 **	(0.05)
Motivation/Pressure (7 elements) ^1^	0.108 **	(0.17)	0.092 **	(0.03)
Conservative governing board (1 = yes) ^1^	−0.022	(0.72)	−0.034	(0.14)
Barriers (12 elements) ^1^	−0.021	(0.15)	0.046 *	(0.03)
Social Inclusion				
Engagement (2 elements, scale 1–3) ^1^	0.126 **	(0.36)	0.047 *	(0.07)
Attitude (5 elements, scale 1–5) ^1^	0.018	(0.13)	0.020	(0.03)
Common vision (3 elements, scale 1–5) ^1^	0.094 **	(0.18)	0.055 *	(0.03)
Child socioeconomic condition				
Percent population under 18 (%) ^2^	0.083 **	(0.08)	−0.023	(0.01)
Population growth under 18 (%) ^2,3^	−0.005	(0.03)	0.017	(0.01)
Poverty rate under 18 (%) ^2^	−0.096 **	(0.03)	−0.001	(0.01)
Demographic structure				
Percent White population (%) ^2^	−0.026	(0.02)	−0.001	(0.00)
Population (ln) ^2^	0.059	(0.31)	0.160 **	(0.06)
Population density (ln) ^2^	0.383 **	(0.21)	0.063 *	(0.04)
Suburb (1 = yes) ^4^	−0.132 **	(1.08)	−0.118 **	(0.21)
Rural (1 = yes) ^4^	−0.042	(1.32)	−0.095 *	(0.25)
Goodness of fit	0.34		0.49	

*n* = 1312 US municipalities and counties. Data sources: ^1^ Planning for all ages survey 2019, ^2^ American Community Survey 2014–2018, ^3^ American Community Survey 2009–2013, ^4^ Census Bureau Delineation files Sept. 2018. Note: ** *p* < 0.05, * *p* < 0.01.
